# The Management of Near-Fatal Hemoptysis with Left Secondary Carinal Y Stent

**DOI:** 10.1155/2014/709369

**Published:** 2014-08-27

**Authors:** Levent Dalar, Cengiz Özdemir, Sinem Sökücü, Levent Karasulu, Sedat Altın

**Affiliations:** ^1^Department of Pulmonary Medicine, School of Medicine, İstanbul Bilim University, Istanbul, Turkey; ^2^Yedikule Chest Diseases and Thoracic Surgery Teaching and Research Hospital, Zeytinburnu, 34760 Istanbul, Turkey

## Abstract

Massive hemoptysis can be a life threatening condition and needs urgent treatment in lung cancer. In the fiberoptic bronchoscopy of a fifty-two-year-old who was admitted with hemoptysis, left upper lobe upper division orifice was seen totally obstructed with a submucosal infiltration. One hour after the mucosal biopsies, massive hemoptysis occurred. Urgent rigid bronchoscopy was performed. The left main bronchus was occluded by sterile gauze. After cleaning of the coagulum patient was intubated and charged to intensive care unit. The next day, rigid bronchoscopy was repeated and the bleeding was observed to continue from the left upper lobe. Removing the gauze, 14 × 10 × 10 mm silicon Y stent was inserted in the left main bronchus after adjustments were made. Bleeding was stopped after insertion of the stent and patient could be extubated. In this case a successful control of hemoptysis was sustained after insertion of a customized silicon stent was presented.

## 1. Introduction

Lung cancer is one of the common causes of massive hemoptysis [1–4].

The treatment of massive hemoptysis has several options including ensuring the safety of the airways, bronchial artery embolization, and surgical treatment. Bronchoscopic methods are frequently used and are efficient both in the diagnosis and in the treatment of hemoptysis [1]. Surgical treatment is not always possible particularly in patients with advanced lung cancer. Endobronchial stent insertions have been published as case reports as an alternative treatment method to control hemoptysis in some selected patients [5–7]. We presented a case who developed massive hemoptysis after diagnostic bronchoscopic procedure and in whom massive hemoptysis was controlled successfully by customized silicone stent application on left secondary carina.

## 2. Case Report

**Figure 1:**
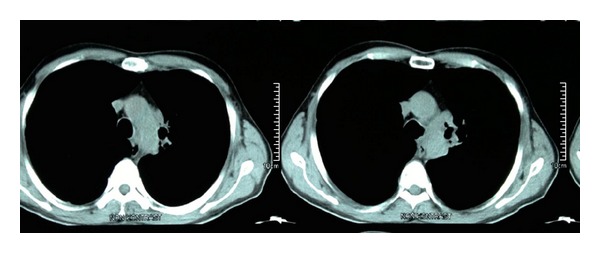
A mass lesion constricting the upper left lobe bronchus with hazy boundaries with the descending aorta observed in the thoracic CT.

**Figure 2:**
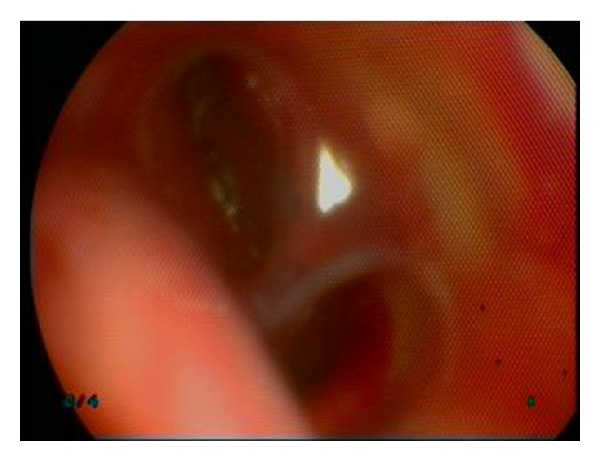
The orifice of the upper left lobe appears to be obstructed by submucosal infiltration bronchoscopically. Following Y stent insertion, the inlet of this lobe is observed to be occluded with the sealed leg and the lower lobe is observed to be clear and functional.

A 52-year-old male patient was admitted with complaints of hoarseness and bloody sputum. The patient has 30 pack/years of smoking history. In thoracic computerized tomography (CT), a mass lesion with irregular contours was observed surrounding aortic arc and the left main bronchus in the mediastinum and causing a loss of calibration in the left pulmonary artery (Figure 1). Fiberoptic bronchoscopy performed under conscious sedation with midazolam revealed mucosal infiltration at the orifice of the upper division of left upper lobe having a tendency for bleeding, almost fully obstructing the orifice. Also lingular segment was almost fully obstructed and constricted at an advanced level. Mucosal biopsies were obtained from these areas and the procedure was terminated by the control of minor bleeding. After approximately one hour, sudden massive hemoptysis developed (about 600 cc in 15 seconds). The patient was urgently taken to the operation room and was intubated by rigid bronchoscope. Fluid replacement, inotropic agents, fresh frozen plasma, and erythrocyte infusion were given. The lumen was observed to be covered with coagulum starting from the trachea inlet. After cleaning the coagulum, active hemorrhage was observed from the entrance of the left main bronchus. The sterile gauze impregnated with epinephrine (0.2%) was placed towards the distal part of the left main bronchus using rigid forceps to occlude the area which origin of the bleeding. In the meantime, cardiac massage was applied for 5 minutes due to cardiac arrest. After stabilization of the patient, the right bronchial tree was carefully cleaned from bleeding residues and bleeding control was ensured. Patient was intubated and transferred to the intensive care unit. The patient was connected to mechanical ventilator under mild sedation for 24 hours. The condition of the patient was stable and approximately 50 cc bleeding occurred through the orotracheal tube in 24 hours. The next day, rigid bronchoscopy both for removing the gauze and for ensuring long-term control over bleeding was done. The gauze at the distal part of the left main bronchus was removed carefully with a forceps. The bleeding was observed to continue massively from the mucosal infiltration area obstructing the upper division. The lesion invaded the mediastinal and main vascular structures so that surgical procedure could not be performed. A silicone Y stent, 14 × 10 × 10 mm in size, was planned to be placed, for bleeding control, in the left main bronchus of the patient who was not suitable for the embolization which we do not have in our hospital and not suitable to be transported to another center due to high risk. The leg of the stent extending to the left main bronchus was closed up to carina level, the inlet of the upper lobe bronchus, by applying bronchial stapler and the leg of the stent extending to left lower lobe bronchus was cut and placed in appropriate size, so that 5 mm remains (Figure 2). After ensuring that bleeding did not continue following placement of the stent, the procedure was terminated. The patient was observed in the intensive care unit for a day. He was extubated at the 4th hour of his follow-up. Pathological examination of biopsy specimens revealed non-small cell lung cancer. The patient receiving palliative chemotherapy concomitant with radiotherapy is followed with stent without hemoptysis in the 3rd month. No complication due to the stent was observed in two sessions of fiberoptic bronchoscopy within this period of time.

## 3. Discussion

Hemoptysis due to lung cancer is observed at a high rate such as 19–32%. Although it is in the form of bloody sputum, it can rarely appear as massive hemoptysis [4]. Life threatening massive hemoptysis may occur due to therapeutic brachytherapy, because of the invasion of bronchial and pulmonary arteries with tumor tissue and development of necrosis in cases with excessive tumor load [7]. So, the massive hemoptysis is seen in patients with lung cancer, usually in advanced disease or in cases receiving radiotherapy.

Bronchial artery embolization, surgical approach, and conservative methods were applied due to the centers capability of the treatment of hemoptysis. Surgical therapy cannot be applied especially for malignant causes and bronchial artery embolization remains the only therapeutic option [1, 8, 9]. Using methods providing a clear airway together with supporting treatments in the management of massive hemoptysis has vital importance. Rigid bronchoscopy in the control of massive hemoptysis can help in the localization of the source of bleeding and for the opening of the airway and assists the application of the interventional bronchoscopic methods. Methods such as rapid absorption of bleeding residues, correct location of the bleeding area, local epinephrine administration, and endobronchial tamponade with balloon are frequently performed through bronchoscopy [1]. Although stent applications are frequently performed in endobronchial treatments done in malignant airway obstructions, experience for bronchoscopic applications in management of hemoptysis is limited to a few cases.

After local control of massive bleeding in this patient, stent was placed for endobronchial tamponade. Chung et al. have reported a case where a covered nitinol stent was placed in the left main bronchus and bleeding was controlled efficiently [6]. In a case of inoperable advanced non-small cell lung cancer reported by Brandes et al., the bleeding emanating from the cavity in the left lower lobe was controlled by placing a Polyflex stent in the lower lobe bronchus and using a coated nitinol Ultraflex stent extending from the left main bronchus to the upper lobe bronchus [5]. In the report published by Lee et al., stent has been applied to 3 cases with lung cancer to manage hemoptysis [7]. The published cases are generally those previously diagnosed with lung cancer and who received oncologic treatment. In our case, massive hemoptysis developed during bronchoscopic biopsy performed for diagnostic procedures. Although minor bleeding is seen due to diagnostic bronchoscopic interventions, massive hemoptysis is seen very rarely. In a study of Jin et al., massive hemoptysis was reported in 19 cases out of 23682 patients who underwent bronchoscopy [10]. After development of massive hemoptysis due to bronchoscopic biopsy, left hilar lesion invaded to the mediastinum is not appropriate for the surgery. Arterial embolization was thought as one of the treatment options for this patient. On the other hand, bronchial artery embolization cannot be applied in our center and the transport of the patient to another center includes very high risk. So, that bronchial artery embolization is not planned.

## 4. Conclusion

Endobronchial stent insertions enable a safe treatment of benign and malignant airway obstructions [11, 12]. We think that reshaped silicone stent applications may be effective particularly in endobronchial tamponade and in isolating the bleeding area in massive hemoptysis cases caused by malign diseases in which surgery and bronchial artery embolization cannot be applied. To our knowledge, this approach has never been done before. We have successfully controlled bleeding, using the local barrier effect of silicone stents at the left secondary carina level. It is planned to remove the stent following the completion of radiotherapy.
